# Upregulation of NDUFAF2 in Lung Adenocarcinoma Is a Novel Independent Prognostic Biomarker

**DOI:** 10.1155/2023/2912968

**Published:** 2023-01-17

**Authors:** Kang Zou, Pengxiang Gao, Xinping Xu, Wei Zhang, Zhenguo Zeng

**Affiliations:** ^1^Department of Critical Care Medicine, Medical Center of Anesthesiology and Pain, The First Affiliated Hospital of Nanchang University, Nanchang City, Jiangxi Province 330006, China; ^2^Department of Critical Care Medicine, The First Affiliated Hospital of Gannan Medical College, Ganzhou City, Jiangxi Province 341000, China; ^3^Department of Respiratory Medicine, The First Affiliated Hospital of Nanchang University, Nanchang City, Jiangxi Province 330006, China; ^4^Jiangxi Institute of Respiratory Diseases, The First Affiliated Hospital of Nanchang University, Nanchang City, Jiangxi Province 330006, China; ^5^Jiangxi Clinical Research Center for Respiratory Diseases, The First Affiliated Hospital of Nanchang University, Nanchang City, Jiangxi Province 330006, China

## Abstract

**Background:**

NADH dehydrogenase (ubiquinone) 1 alpha subcomplex assembly factor 2 (NDUFAF2) acts as a molecular chaperone for the assembly of complex I on the mitochondrial membrane, which is involved in the transfer of electrons in the respiratory chain. However, whether NDUFAF2 plays a role in lung adenocarcinoma (LUAD) is largely unexplored.

**Methods:**

Expression profiles were obtained from the TCGA and GEO databases and integrated via R3.6.3 and several bioinformatics platforms. Western blotting analysis and immunohistochemistry staining were used to examine the expressions of NDUFAF2 in clinical samples. Moreover, the diagnostic and prognostic value of NDUFAF2 expression level was also assessed. GO, KEGG, and gene set enrichment analysis (GSEA) were adopted to investigate NDUFAF2-related molecular functions, signaling pathways, and life activity processes.

**Results:**

NDUFAF2 was predominantly expressed in LUAD, and it is identified as a promising biomarker in the diagnosis of LUAD and its prognostic prediction. Overexpression of NDUFAF2 was correlated with N stage, T stage, and pathologic stage in LUAD, leading to worse overall survival (OS). Besides, the level of NDUFAF2 was independently associated with OS through a multivariate Cox analysis (HR = 1.538, 95% (1.086-2.177), *P* = 0.015). GO analysis revealed enrichment in innate immune response in mucosa and mucosal immune response, and GSEA indicated enrichment in G2_M_checkpoints, DNA replication, diseases of mitotic cell cycle, retinoblastoma gene in cancer, cell cycle pathway, and cell cycle. Furthermore, the expression level of NDUFAF2 was negatively correlated with infiltration levels of Tem, Tcm, NK CD56^bright^ cells, and B cells. In contrast, the expression level of NDUFAF2 was positively correlated with the infiltration level of DCs and Th2 cells in LUAD patients.

**Conclusions:**

Collectively, NDUFAF2 is a promising independent prognostic biomarker and target in LUAD. In addition, NDUFAF2 might affect the prognosis of LUAD via DNA replication, diseases of mitotic cell cycle, cell cycle pathway, and cell cycle.

## 1. Introduction

Lung adenocarcinoma (LUAD) is a heavy burden on public health worldwide. According to the report of cancer incidence and mortality by GLOBOCAN 2020, LUAD ranks second in all commonly diagnosed cancers (11.4%), and it causes 18% of the cancer-related death in 2020. The 5-year survival of LUAD is approximately 20% in recent years [1], and the up-to-date histopathologic classification shows that LUAD comprises most of all pulmonary malignancies, accounting for approximately 40% of lung malignancies [2]. Notable changes in lung cancer epidemiology, therapeutic intervention, and prevention have occurred in the last few decades owing to the improvement of targeted therapies and immunotherapy. More novel methods are currently being investigated, such as immune checkpoint blockade, oncolytic viruses, stem cell-based medicinal products, cytokine derivatives, CD3-bispecific antibodies, vaccine platforms, and adoptive tumor cell therapy [3]. With further research in the field of oncology, the tumor microenvironment (TME) is now generally appreciated to play an indispensable role in tumorigenesis and malignant progression [4]. Until now, the relative prognosis of LUAD remains unsatisfactory. It is imperative that we continue to identify new diagnostic methods and targets.

At present, it has become a common method to search for lung adenocarcinoma prognostic markers through bioinformatics. By combining high-throughput information of omics with clinical information of patients, effective prognostic markers can be screened, and then, markers can be further analyzed and studied [5–7]. Finally, this makes it possible for them to become reliable indicators [8]. Through the analysis of multiomics outcome (Supplementary Materials) from LUAD samples [7], we reported that the level of NDUFAF2 in the tumor tissues is higher than that in adjacent nontumor tissues. Therefore, we speculated that NDUFAF2 could be a promising prognostic indicator in the diagnosis and treatment of LUAD.

In the traditional view, NDUFAF2 is a complex I assembly factor in mitochondria. Functional loss of genes encoding complex I subunits greatly contributes to abnormal function of the respiratory chain, leading to various clinical phenotypes from early onset lethal symptoms to adult-onset exercise intolerance, such as Leigh's syndrome [9]. The homozygous substitution in NDUFAF2 is definitely the pathogenic mutation, which results in the lack of complex I in the fibroblasts of the patient, and the expression and functional activity of complex I can almost entirely be rescued by complementation of the patient's fibroblasts with the baculovirus [10]. Furthermore, depletion of NDUFAF2 in human SK-N-MC neuroblastoma cells can inhibit cell proliferation [9]. Besides, NDUFAF2 is also known as “mimitin” identified in the screening of Myc-regulated genes. It is upregulated by c-Myc in some cell lines, such as glioblastoma T98G, ESCC TE-11, and human embryonic lung (HEL) cells, and specific inhibition of mimitin reduces cell proliferation [11].

Further analysis was carried out with regard to NDUFAF2 in this study. We explored the predictive value of level of NDUFAF2 in LUAD based on clinical information obtained from The Cancer Genome Atlas (TCGA). In addition, Gene Expression Profiling Interactive Analysis (GEPIA) and COX regression analysis were carried out. Simultaneously, the proportions of various tumor-infiltrating immune cells (TIICs) in different TMEs were adopted to explore the correlation between NDUFAF2 and TIICs according to CIBERSORT.

To further explore the potential roles of NDUFAF2, the analyses of biological pathways included in the LUAD tumorigenesis-related NDUFAF2 regulatory network were conducted using gene set enrichment analysis (GSEA) and Gene Ontology (GO) analysis. Furthermore, the probable correlation between NDUFAF2 and TIICs in a variety of TMEs was assessed using single-sample GSEA (ssGSEA). NDUFAF2 might play a key role in cancer cell proliferation, which could be used as a new independent prognostic marker, and its expression level was correlated with immune infiltrates in LUAD.

## 2. Material and Methods

### 2.1. Clinical Specimens

Six pairs of fresh LUAD tissues and adjacent nontumor lung tissues were collected from patients who underwent thoracic surgical treatment. The data from December 2020 to April 2021 were obtained from the First Affiliated Hospital of Nanchang University. The collected tissue specimens were immediately preserved in liquid nitrogen and then at -80°C. Our current study was approved by the ethics committee of the First Affiliated Hospital of Nanchang University (No. 2022-1-018). Signed written informed consents were provided by all six LUAD patients, and they did not undergo any chemotherapy or radiation therapy before surgery or receive other special treatment prior to the operation. Moreover, these patients had no other malignancies. The flow diagram of this study is shown in Supplementary Figure 1.

### 2.2. Acquisition of Detailed Patient Data

The mRNA profiles including 513 LUAD specimens and 59 adjacent nontumor tissue specimens (workflow typed: TPM), clinicopathological data, and other data were obtained from the TCGA database (https://cancergenome.nih.gov) in March 2022. In addition, an independent external dataset was extracted, and the GSE116959 including 57 LUAD specimens and 11 nontumor tissue specimens acquired from the GEO database (https://www.ncbi.nlm.nih.gov/geo/) was adopted to confirm the differential expression of NDUFAF2. Furthermore, the expression of NDUFAF2 at the mRNA level between LUAD and nontumor tissues was compared using the GEPIA (http://gepia.cancer-pku.cn). Moreover, the expression of NDUFAF2 at the protein level between the LUAD tissues and nontumor tissues was analyzed using the Human Protein Atlas (HPA) (https://www.proteinatlas.org) and the UALCAN web (http://ualcan.path.uab.edu/index.html). In the present study, our databases were in agreement with the publication guidelines offered by the online database, and approval by ethics committee and informed consent were waived.

### 2.3. GO/Kyoto Encyclopedia of Genes and Genomes (KEGG) Enrichment Analysis and GSEA

KEGG pathway enrichment analysis and GO term analysis of the differentially expressed genes (DEGs) were carried out using the XIANTAO web (http://www.xiantao.love) based on the cluster Profiler R package (v3.18.0). GSEA was applied to explore the biological functions and enrichment pathways correlated with NDUFAF2 in LUAD. For each analysis, gene set permutation was performed 1,000 times. Normalized enrichment score (NES) > 1, gene sets with false discovery rate (FDR) *q* value < 0.25, and nominal (NOM) *P* value < 0.05 were regarded as significant.

### 2.4. Tumor Immune Infiltration Analysis

The expression level of NDUFAF2 in 24 types of tumor immune cells was calculated using the ssGSEA with the XIANTAO platform based on the GSVA package (version 1.36.3) [12, 13].

### 2.5. Cell Lines and Culture Condition

Six types of human LUAD cell lines (PC9, H358, A549, HCC827, H1299, and H1975) and pulmonary epithelial cell line (BEAS-2B) were provided by the Procell Life Science &Technology Co., Ltd. (Wuhan, China). These cells were maintained in DMEM (Gibco) or RPMI-1640 (Hyclone) supplemented with 10% fetal bovine serum (FBS), 1% streptomycin, and 1% penicillin at 37°C in a humidified atmosphere containing 5% CO_2_.

### 2.6. Western Blotting Analysis

The samples of cells and tissues were homogenized in RIPA lysis buffer with 1% protease inhibitor, 1% phosphatase inhibitor, and 1% PMSF (Applygen, Beijing, China). The same amount of proteins was analyzed by 12% sodium dodecyl sulfate-polyacrylamide gel electrophoresis (SDS-PAGE) (Servicebio, Wuhan, China) and then electrotransferred onto 0.2 *μ*m polyvinylidene fluoride (PVDF) membranes (Millipore, Billerica, MA, USA). The membrane was incubated with the rabbit monoclonal anti-mimitin C-terminal antibody (1: 1,000, ab192267; Abcam, MA, USA) and the mouse anti-*β*-actin antibody (1: 5,000, 66009-1-Ig; Proteintech, Wuhan, China) overnight at 4°C. Subsequently, the membrane was incubated with horseradish peroxidase- (HRP-) conjugated goat anti-rabbit IgG (H+L) (1 : 5,000; 111-035-003, Jackson, Shanghai, China) or goat anti-mouse IgG (H+L) (1 : 5,000; 115-035-003, Jackson, Shanghai, China) for 2 h at room temperature. The stained bands were visualized and analyzed using ImageJ software (version1.51, National Institutes of Health, USA).

### 2.7. Immunohistochemistry (IHC) Staining

Paraffin-embedded postoperative human LUAD tissues and nontumor tissues were prepared for IHC staining. The tissue block was sectioned into 4 *μ*m slices. Subsequently, the tissue sections were incubated with a primary antibody against mimitin (1 : 100 dilution, ab192267; Abcam, MA, USA) overnight at 4°C. The slides were then washed with PBS twice, followed by incubation with an HRP-conjugated goat anti-rabbit/mouse secondary antibody (PV6000; ZSGB-Biotech, Beijing, China) at room temperature for 20 min. IHC staining was carried out using 3,3′-diaminobenzidine (ZSGB-Biotech, Beijing, China), and the slides were counterstained with hematoxylin which was from Beyotime (Shanghai, China). The stained cells were observed under a microscope.

### 2.8. Statistical Analysis

The software R (version 3.6.3) was used to perform all statistical analysis, and R package ggplot2 (version: 3.3.3) was used to analyze the expression profiles based on the XIANTAO platform. Differences between the two groups were compared using the Wilcoxon rank-sum test, and the analysis of variance (ANOVA) was adopted for multigroup comparisons, followed by a Student's *t*-test. The receiver operating characteristic (ROC) curve was applied to investigate the diagnostic value, and a nomogram was employed to assess the predictive value. The death risk, including age, gender, TNM stage, pathologic stage, smoking history, and NDUFAF2 expression, was assessed using univariate and multivariate Cox regression analyses. All tests were two-sided, and a *P* value < 0.05 was treated as statistically significant. ^∗^, ^∗∗^, and ^∗∗∗^ represent *P* < 0.05, *P* < 0.01, and *P* < 0.001, respectively, in the analysis.

## 3. Results

### 3.1. Clinical Characteristics of Patients

Table 1 lists the association of the NDUFAF2 expression levels with the clinical characteristics of LUAD patients. The clinical parameters of 513 LUAD patients were downloaded from the TCGA database in March 2022. The baseline clinicopathologic characteristics included age, gender, pathologic stage, TNM stage, smoking history, and overall survival (OS). Among the LUAD patients, 276 were females (53.8%), and there were 248 patients aged less than 65 (48.2%). These data illustrated that the high expression level of NDUFAF2 was remarkably associated with the pathologic stage (*P* < 0.001), T stage (*P* < 0.001), N stage (*P* < 0.001), and OS (*P* < 0.001).

### 3.2. The Overexpression of NDUFAF2 of LUAD Patients

**Figure 1:**
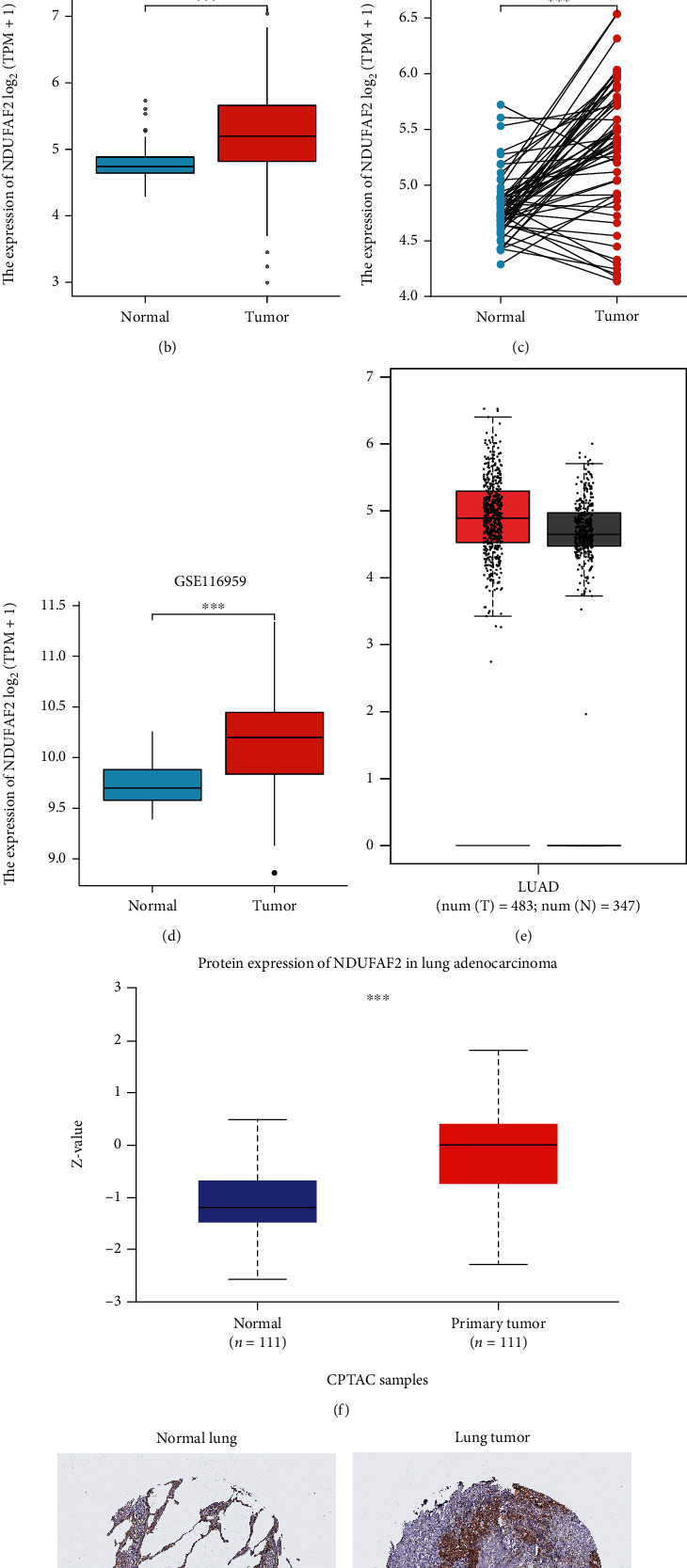
The expression of NDUFAF2 at the mRNA and protein levels in LUAD tissues. (a) The expression of NDUFAF2 in different tumors in the TCGA database. (b) NDUFAF2 expression in unpaired normal lung tissues and LUAD from the TCGA database. (c) NDUFAF2 expression in paired tissues using the TCGA database. (d) The high expression of NDUFAF2 was testified in LUAD from the GEO database. (e) The high expression in LUAD was confirmed using the match TCGA normal and GTEx data based on GEPIA platform. (f) The UALCAN was used to detect the protein distribution of NDUFAF2 in LUAD patients. (g, h) The HPA showed the expression of NDUFAF2 at the protein level in LUAD tissues and normal lung tissues using IHC staining. ^∗∗∗^ indicates *P* < 0.001, ^∗∗^ indicates *P* < 0.01, and ^∗^ indicates *P* < 0.05.

**Figure 2:**
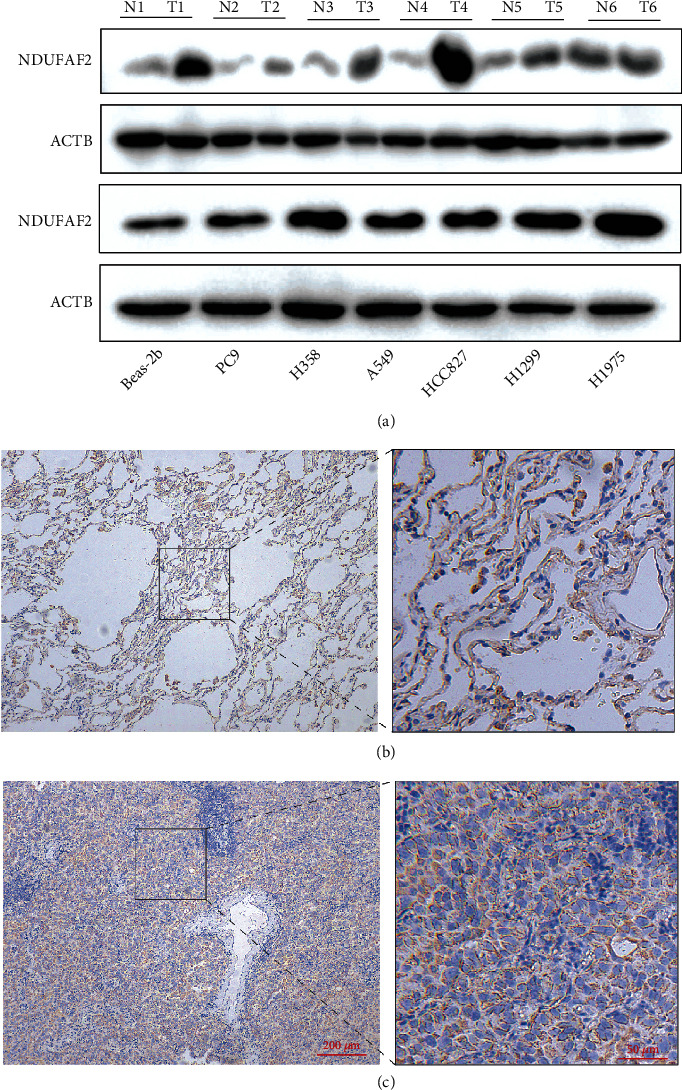
The expression of NDUFAF2 at the protein level in LUAD tissues was confirmed using clinical tissue and NSCLC cell lines. (a) Validation of the expression level of NDUFAF2 in LUAD and paired adjacent lung tissues was conducted using western blotting analysis. (b) Western blotting analysis was used to detect the protein expression level of NDUFAF2 in different NSCLC cell lines and normal lung epithelial cell line 2B. (c) IHC staining of NDUFAF2 was performed in adenocarcinoma tissue and paired adjacent normal lung tissue from typical clinical specimens. Representative images are shown. Scale bar, left, 200 *μ*m; right, 50 *μ*m.

To identify the expression pattern of NDUFAF2 at the mRNA level in tumor and nontumor lung tissues, we analyzed the expression level of NDUFAF2 in various tumors and normal tissues using TCGA database. The expression level of NDUFAF2 was markedly greater in tumor tissues, such as adrenocortical carcinoma (ACC), cholangiocarcinoma (CHOL), diffuse large B cell lymphoma (DLBCL), glioblastoma multiforme (GBM), kidney renal papillary cell carcinoma (KIRP), kidney renal clear cell carcinoma (KIRC), low-grade glioma (LGG), LUAD, liver hepatocellular carcinoma (LIHC), prostate adenocarcinoma (PRAD), pancreas adenocarcinoma (PAAD), skin cutaneous melanoma (SKCM), rectal adenocarcinoma (READ), thymoma (THYM), stomach adenocarcinoma (STAD), and thyroid carcinoma (THCA). In addition, a lower expression of NDUFAF2 was observed in breast invasive carcinoma (BRCA), cervical squamous cell carcinoma and endocervical (CESC), bladder urothelial carcinoma (BLCA), esophageal carcinoma (ESCA), acute myeloid leukemia (LAML), kidney chromophobe carcinoma (KICH), ovarian serous cystadenocarcinoma (OV), and testicular germ cell tumors (TGCT) (Figure 1(a)). Moreover, the average expression level of NDUFAF2 in LUAD tissues was remarkably greater compared with nontumor tissues (*P* < 0.001, Figure 1(b)). Similar findings were confirmed in paired LUAD tissues and adjacent nontumor tissues (*P* < 0.001, Figure 1(c)). Similar results showing the higher expression of NDUFAF2 at the mRNA level in LUAD were obtained from the GEO database using GSE116959 datasets (Figure 1(d)). Its high expression in LUAD was also confirmed using the matched TCGA and GTEx data based on the GEPIA platform (Figure 1(e)). We also evaluated the expression of NDUFAF2 at the protein level in LUAD and adjacent nontumor tissues using the UALCAN web and HPA database. The expression of NDUFAF2 at the protein level was higher in LUAD compared with adjacent nontumor tissues (Figure 1(f)). Moreover, its expression was low in type II alveolar cells and medium in macrophages, while it was not detected in type I alveolar cells and endothelial cells using HPA054776 antibodies (Figure 1(g)). In contrast, high expression of NDUFAF2 at the protein level was detected in LUAD tissues using the HPA database, showing high staining intensity in lung tumor cells using HPA054776 antibodies (Figure 1(h)). Furthermore, we selected six pairs of clinical patient samples from our cohort. Western blotting analysis indicated that the expression of NDUFAF2 was higher in LUAD compared with normal lung tissues (Figure 2(a)). In addition, in vitro experiments revealed that the expression of NDUFAF2 at the protein level in NSCLC cell lines, including PC9, H358, A549, HCC827, H1299, and H1975 cells, was markedly greater compared with BEAS-2B cells (Figure 2(b)). IHC staining of NDUFAF2 was carried out in LUAD tissues and adjacent nontumor tissues. Our results showed that the expression of NDUFAF2 in LUAD was greater compared with adjacent nontumor tissues (Figure 2(c)). Taken together, these findings suggested that the expression of NDUFAF2 at both the mRNA and protein levels was increased in LUAD tissues.

### 3.3. Correlation of NDUFAF2 Expression with Clinical Features in LUAD Patients

**Figure 3:**
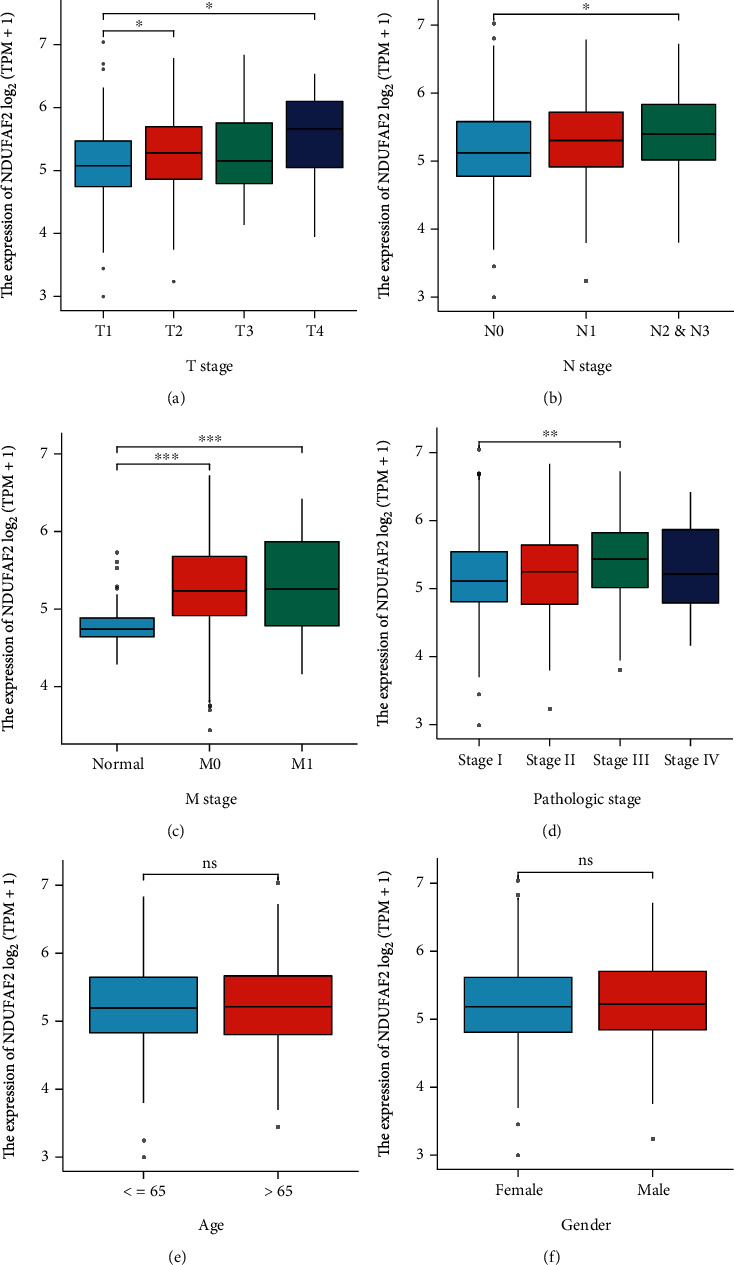
Correlation between NDUFAF2 mRNA expression and clinical pathological characteristics based on TCGA cohort. (a) T stage, (b) N stage, (c) M stage, (d) pathologic stage, (e) age, and (F) gender. ns: no significance. ^∗∗∗^ indicates *P* < 0.001, ^∗∗^ indicates *P* < 0.01, and ^∗^ indicates *P* < 0.05.

To further reveal the correlation of NDUFAF2 expression with clinicopathologic features in LUAD patients, the expression pattern of NDUFAF2 at the mRNA level in various clinical categories was analyzed using the TCGA database. We found that the high expression of NDUFAF2 was remarkably associated with the N stage, T stage, and pathologic stage, but not age and gender (Figure 3).

### 3.4. Analysis of Survival and Subgroup Analysis

**Figure 4:**
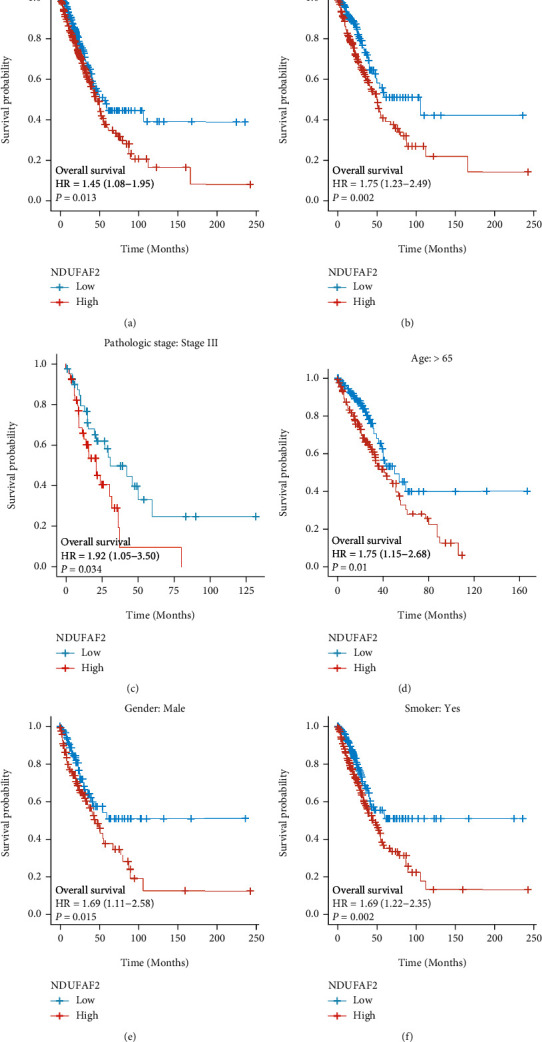
The Kaplan-Meier survival curve analysis of the prognostic significance of the mRNA level of high and low expression of NDUFAF2 in LUAD based on the TCGA database. (a) The Kaplan-Meier estimates the OS. (b–f) Subgroup analysis for (b) M0 stage, (c) pathologic stage III, (d) age over 65 years, (e) male, and (f) smoking history.

The Kaplan-Meier survival analysis was employed in the TCGA cohort to understand whether the expression level of NDUFAF2 affected the survival of patients.  Figure 4(a) reveals that the high expression level of NDUFAF2 was significantly correlated with the poor OS of LUAD patients (HR = 1.45, *P* = 0.013). Further subgroup analysis indicated that a high expression level of NDUFAF2 was related to a poor prognosis of LUAD in the following subgroups: M0 stage (HR = 1.75, *P* = 0.002), pathologic stage III (HR = 1.92, *P* = 0.034), age over 65 years (HR = 1.75, *P* = 0.01), male (HR = 1.69, *P* = 0.015), and smoking history (HR = 1.69, *P* = 0.002). These data are shown in Figures 4(b)–4(f).

### 3.5. NDUFAF2 Is an Independent Risk Factor for OS

The association of NDUFAF2 with the clinical features in LUAD was assessed. High expression level of NDUFAF2 was associated with a poor OS, which was significantly correlated with the pathologic stage and TNM stage (Table 2). Univariate Cox regression analysis showed that the high expression level of NDUFAF2 was markedly related to a poor OS (HR = 1.453, 95%CI = 1.081 ~ 1.953, *P* = 0.013). Table 2 implies that NDUFAF2 was an independent risk factor for OS in LUAD patients (HR = 1.538, 95%CI = 1.086 ~ 2.177, *P* = 0.015), supported by the multivariate Cox regression analysis.

### 3.6. Diagnostic and Predictive Values of NDUFAF2 Expression in LUAD

**Figure 5:**
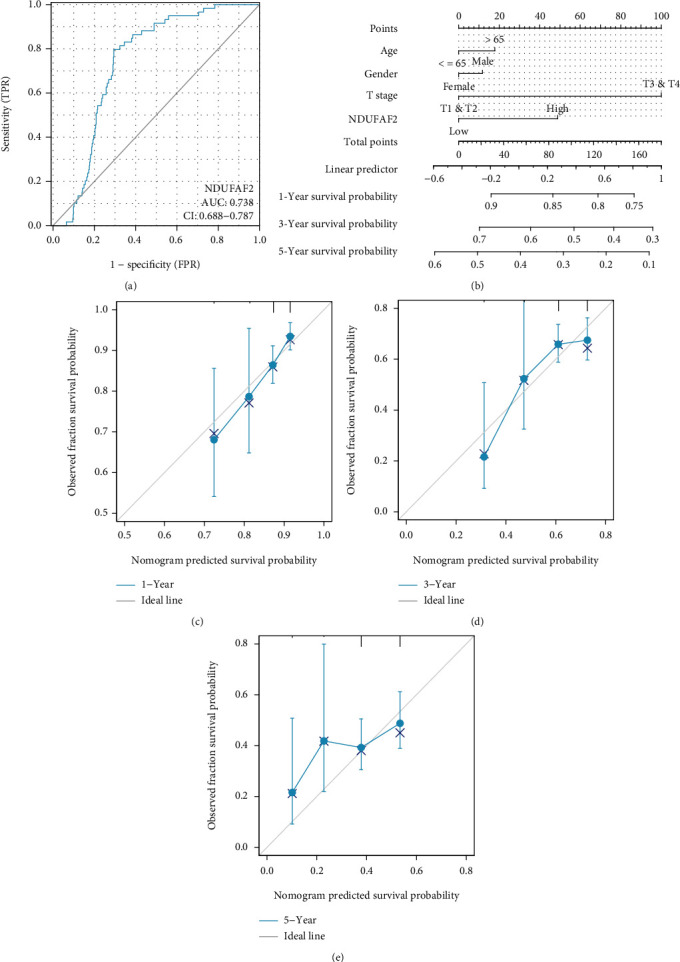
Diagnostic and predictive value of NDUFAF2 expression in LUAD. (a) ROC curve analysis showed that NDUFAF2 had an AUC value of 0.738 when discriminating LUAD tissues from normal lung tissue. (b) A nomogram survival prediction chart for predicting the 1-year, 3-year, and 5-year OS probability. (c–e) Calibration curve of the nomogram.

ROC curve was adopted to analyze the diagnostic value of NDUFAF2 expression level in LUAD patients. The area under the ROC curve (AUC) was 0.738 (Figure 5(a)). Moreover, the AUC value of NDUFAF2 was 0.738 when discriminating LUAD tissues from healthy lung tissues or adjacent nontumor tissues. To detect the predictive value of NDUFAF2 expression level in LUAD, we constructed a nomogram according to the expression profile of NDUFAF2 in the TCGA database. The clinical variables combined with multivariate Cox analysis results and frequent clinical variables age and gender were selected to establish a nomogram to predict the 1-year, 3-year, and 5-year survival probabilities (Figure 5(b)). The nomogram was calibrated with the calibration curve (Figures 5(c)–5(e)).

### 3.7. GO and KEGG Analyses and GSEA

**Figure 6:**
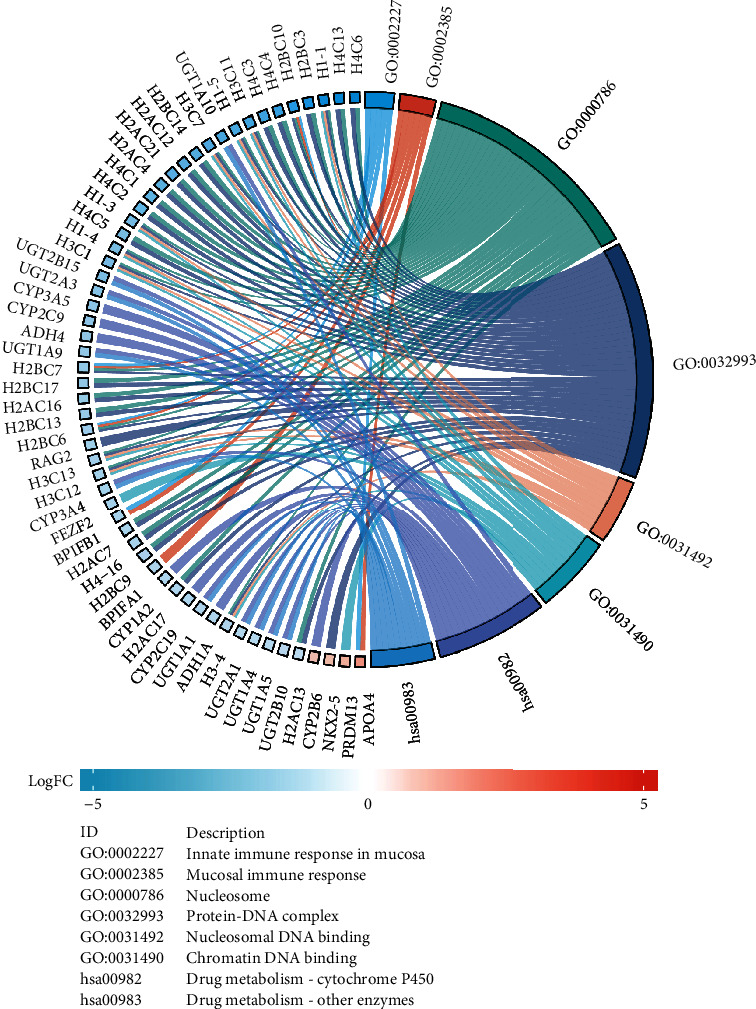
GO and KEGG functional and pathway enrichment analyses of NDUFAF2 in LUAD using a chord diagram.

**Figure 7:**
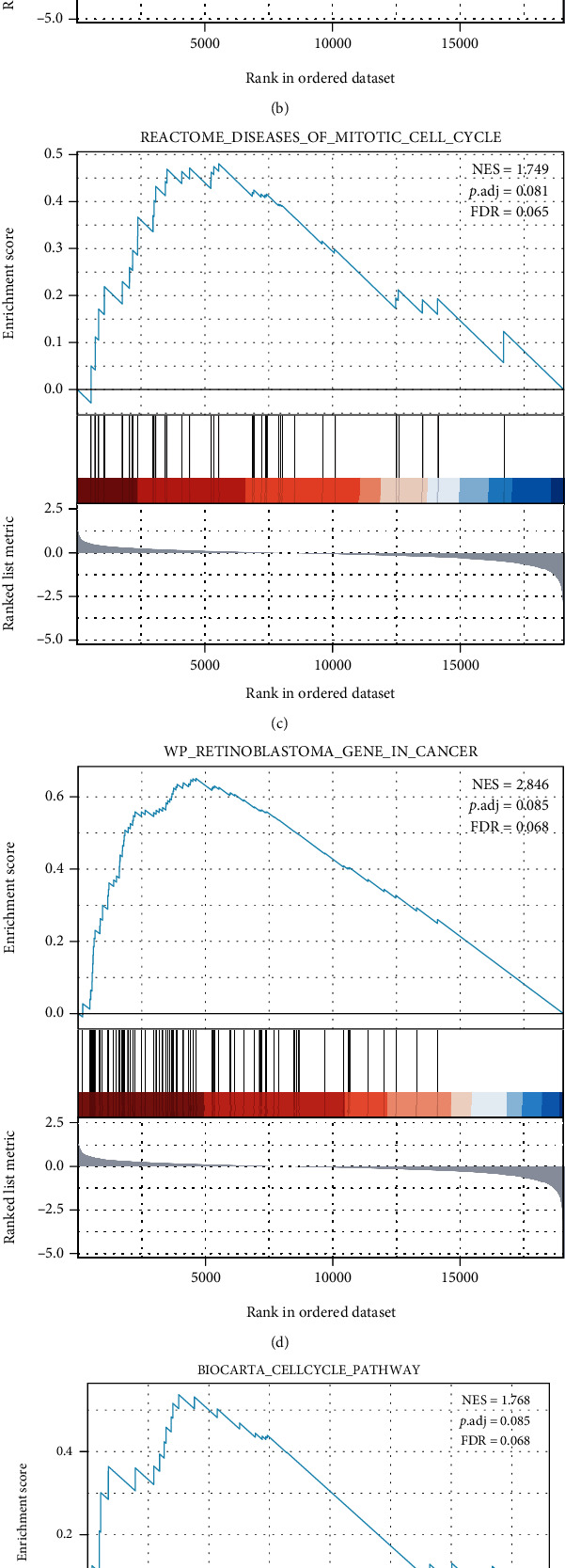
GSEA functional and pathway enrichment analyses of all genes related to NDUFAF2 in LUAD. (a–f) GSEA functional and pathway enrichment analyses of NDUFAF2 in LUAD enrichment in G2_M_checkpoints (a), DNA replication (b), diseases of mitotic cell cycle (c), retinoblastoma gene in cancer (d), cell cycle pathway (e), and cell cycle (f). NES represents normalized enrichment score; NOM represents nominal; FDR represents false discovery rate. NOM *P* value < 0.05, gene sets with |NES| > 1, and FDR *q* value < 0.25 were treated as significantly enriched.

DEGs were identified according to the expression of NDUFAF2. The potential function of NDUFAF2 was predicted based on the enriched GO terms and KEGG pathway analysis (Figure 6). We discovered that these NDUFAF2-associated genes were mainly enriched in GO : 0002227 innate immune response in mucosa, GO : 0002385 mucosal immune response, GO : 0000786 nucleosome, GO : 0032993 protein-DNA complex, GO : 0031492 nucleosomal DNA binding, GO : 0031490 chromatin DNA binding, hsa00982 drug metabolism-cytochrome P450, and hsa00983 drug metabolism-other enzymes. We, respectively, analyzed the NDUFAF2 expression in LUAD enrichment in G2_M_checkpoints, DNA replication, diseases of mitotic cell cycle, retinoblastoma gene in cancer, cell cycle pathway, and cell cycle using GSEA (Figures 7(a)–7(f)). More information on function and pathway enrichment is shown in Table 3.

### 3.8. The Correlation of NDUFAF2 Expression with Cell Cycle Regulatory Genes

The GSEA implied that NDUFAF2 was markedly enriched in the diseases of mitotic cell cycle, cell cycle, and cell cycle pathway. Thus, we further analyzed the association of NDUFAF2 with cell cycle regulatory genes. Our data showed that the expression of NDUFAF2 was positively correlated with several cell cycle regulatory genes, such as CCNA2, CCNB1, CCNB2, CDC6, CDC20, CDC25A, CDC25C, CDC45, CHEK1, MCM2, MCM6, PCNA, PLK1, PTTG1, and MCM4 (Supplementary Figure 2).

### 3.9. NDUFAF2 Expression Is Associated with Immune Cell Infiltration

**Figure 8:**
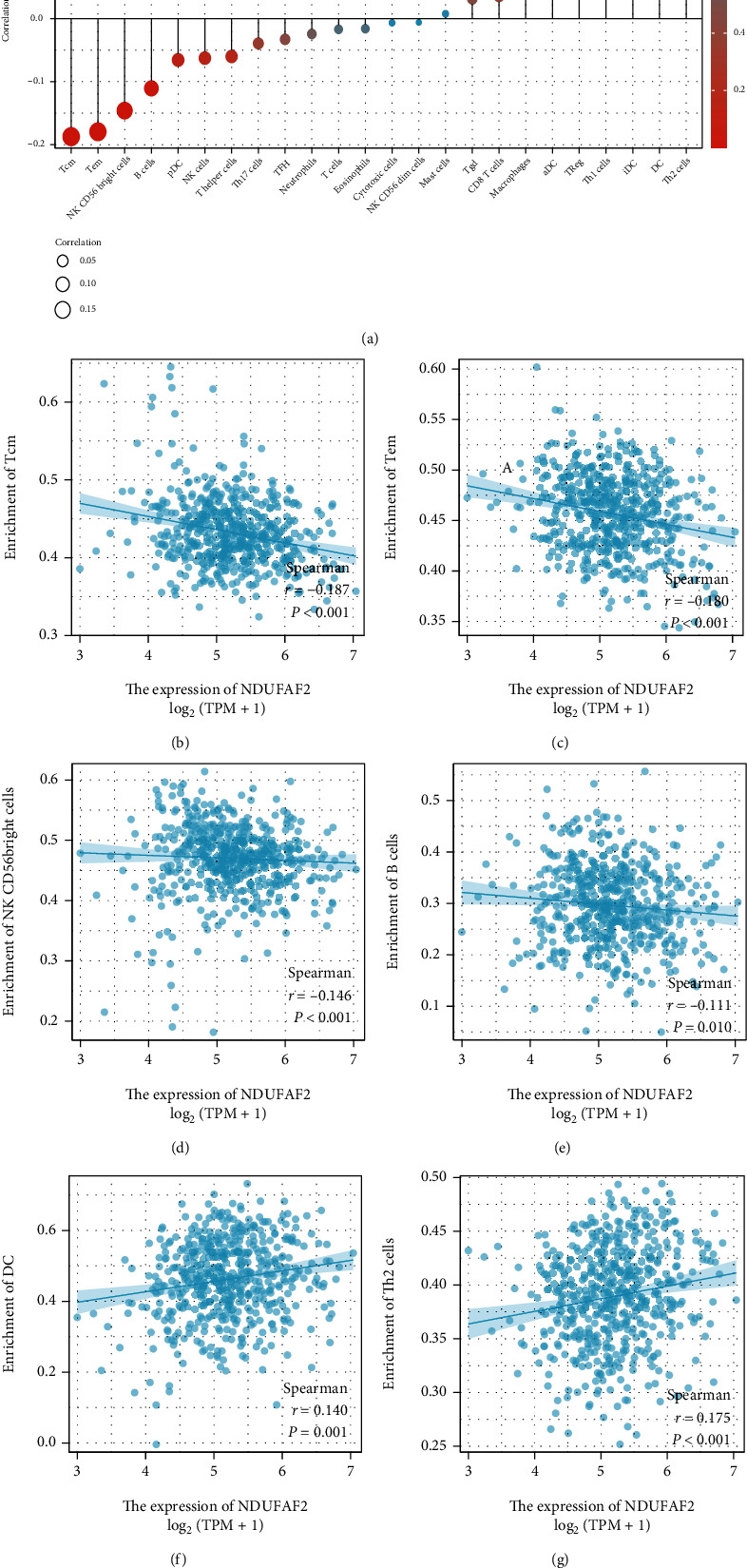
The correlation of NDUFAF2 mRNA expression with the immune infiltration in LUAD using the TCGA database. (a) The correlations for all 24 immune cell types. (b–e) The NDUFAF2 expression was negatively correlated with enrichment of the Tem, Tcm, NK CD56^bright^ cells, and B cells. (f, g) The expression of NDUFAF2 was positively related to the infiltration level of DCs and Th2 cells. iDC: immature DC; Tem: T effector memory; TFH: T follicular helper; Tgd: T gamma delta; pDC: plasmacytoid DC; aDC: activated DC; Tcm: T central memory.

To clarify the association of NDUFAF2 expression level with tumor immune response, we further performed an enrichment analysis of the LUAD TME using ssGSEA.  Figure 8 shows that the Tem, Tcm, B cells, and CD56^bright^ NK cells were negatively associated with the expression level of NDUFAF2. The dendritic cells (DCs) and Th2 cells were positively correlated with the expression level of NDUFAF2. All the above-mentioned findings suggested that upregulation of NDUFAF2 was concomitant with low immune infiltration, leading to a worse OS.

## 4. Discussion

It is well known that NDUFAF2 acts as a mitochondrial complex I assembly factor, and its biological function is conserved from fungi to mammals. The deficiency of NDUFAF2 due to null mutations does not absolutely restrain the assembly or activity of complex I, although it contributes to the development of a progressive encephalopathy [14]. Depletion of NDUFAF2 can impair cell respiration and ATP turnover, increase the output of reactive oxygen species (ROS), and cause oxidative damage, resulting in mitochondrial DNA damage [9]. Some studies have shown that depletion of NDUFAF2 can affect the growth of some cells, such as HEL cells [11]. Abnormality of NDUFAF2 such as mutations causes mitochondrial complex I deficiency [10, 15], mitochondrial complex I is very important in tumor metabolism, and defective NADPH production in mitochondrial disease complex I leads to inflammation and cell death [16]. Mitochondrial complex I donates electrons to ubiquinone, which results in the generation of ubiquinol and the regeneration of the NAD+ and FAD cofactors; simultaneously, the mitochondrial electron transport chain (ETC) is necessary for tumor growth [17]. Human lung tumors display robust glucose oxidation by mitochondria [18]. We speculated that NDUFAF2 mainly affects mitochondrial complex I to influence the progression of lung adenocarcinoma. Complex I is the first enzyme of the mitochondrial respiratory chain and consists of 45 subunits in humans; it is one of the largest known multisubunit membrane protein complexes [19]. Complex I is assembled via a series of intermediate assembly modules and requires the involvement of more than 10 known assembly factors; some assembly factors are homologous to structural subunits of complex I and serve as their “place holders” in assembly intermediates; for instance, NDUFAF2 is a homologue of the NDUFA12 subunit [20]. In NDUFA12-knockout cells, the NDUFAF2 substituted for its paralogue NDUFA12, leading to complex I appearing fully assembled [19, 21]. Tumor growth is clearly affected by function by mitochondrial complex I [17].

In our present study, through bioinformatics of NDUFAF2 expression in different tissues, we noticed that the expression of NDUFAF2 was remarkably greater in LUAD compared with adjacent nontumor tissues. IHC staining images acquired from the HPA also supported this conclusion. We externally validated the differences at the protein and pathological levels via western blotting analysis and IHC analysis. Furthermore, the expression of NDUFAF2 was significantly higher in the lung cancer cell lines. A prognostic nomogram was also established, including age, gender, T stage, and the expression of NDUFAF2, which could be used to enhance the accuracy in identifying patients at high risk. A higher point on the nomogram indicated a worse clinical outcome.

The GO and KEGG analyses and GSEA were carried out to deeply investigate the biological functions of NDUFAF2. GSEA showed that G2M checkpoints, DNA replication, cell cycle, and DNA damage response were differentially enriched with the NUDFAF2 expression phenotype, and KEGG/GO indicated that pathways in innate immune response in mucosa, mucosal immune response, nucleosome protein-DNA complex, and nucleosomal DNA binding were mainly enriched. Uncontrolled, unlimited, and accelerated proliferation is one of the primary life behaviors of tumor cells [22]. Tumor cells can cease the cell cycle to repair DNA damage through the G2-M checkpoint. Previous studies have shown the function of WEE1 kinase in mediating the G2/M cell cycle checkpoint, and it is hypothesized that inhibition of WEE1 is most active in a TP53-mutated background, where cells lose the G1/S cell cycle checkpoint, leading to enhanced dependence upon the G2/M checkpoint. The antitumor drug based on WEE1 inhibition has completed phase II clinical trial [23, 24].

Additionally, we analyzed the connection of the NDUFAF2 expression level and immune cells in LUAD by using immune cell infiltration datasets from CIBERSORT. We observed that there was a positive correlation of the expression level of NDUFAF2 with the infiltration of immunosuppressive cells, such as Th2 cells, while there was a negative correlation of its expression level with immune killer cells, such as effector memory T cells (Tem) and central memory T cells (Tcm). This finding was similar to the results of the immune landscape from preneoplasia to invasive LUAD, in which tumors are found to be Th2-skewed [25]. Th2 cells release IL-5, IL-4, and IL-13, resulting in tumor cell growth and metastasis. TH1/TH2 ratio is important in the antitumor immune response, indicating that strengthening the TH1 response and inhibiting the TH2 activation and immunity-related cytokines may concurrently help eliminate disseminated tumor cells, preventing cancer metastasis and recurrence [26]. Meanwhile, Tcm cells possess higher persistence and antitumor immunity than Tem cells and effector T (Teff) cells [27]. Besides, the Tcm/Teff ratio has been recommended as a predictive biological indicator of response to the treatment of checkpoint inhibitors in NSCLC patients [28]. The drawback of our work here was that only a small sample was validated, and the actual function of NDUFAFA2 in LUAD needs to be further validated. In summary, the functionality of NDUFAF2 was multiaspect. It played a key role in predicting the prognosis of LUAD. However, the detailed effects and mechanism of NDUFAF2 in LUAD remain to be further explored in the future study.

## 5. Conclusions

Our research revealed that NDUFAF2 was predominantly expressed in LUAD, and its expression played a critical role in the diagnosis and prognosis of LUAD. In human LUAD, the expression of NDUFAF2 was upregulated, and overexpression of NDUFAF2 was correlated with N stage, T stage, and pathologic stage in LUAD, leading to a worse OS. Moreover, the expression of NDUFAF2 was an independent prognostic biomarker in LUAD. In addition, NDUFAF2 might affect the prognosis of LUAD via DNA replication, diseases of mitotic cell cycle, cell cycle pathway, and cell cycle. Furthermore, upregulation of NDUFAF2 in LUAD was positively correlated with tumor immune infiltration of DC and Th2 cells. Our current findings provided valuable insights into the potential therapeutic regimens of LUAD.

## Figures and Tables

**Table 1:** Correlation between NDUFAF2 expression and baseline clinicopathologic characteristics of LUAD patients from the TCGA database.

Characteristic	Low expression of NDUFAF2	High expression of NDUFAF2	*P*
*n*	256	257	
Age, *n* (%)			0.928
≤65	125 (24.3%)	123 (23.9%)	
>65	131 (25.7%)	134 (26.1%)	
Gender, *n* (%)			0.892
Female	139 (27.1%)	137 (26.7%)	
Male	117 (22.8%)	120 (23.4%)	
Pathologic stage, *n* (%)			<0.001
Stage I	156 (30.9%)	118 (23.4%)	
Stage II	57 (11.3%)	64 (12.7%)	
Stage III	26 (5.1%)	58 (11.5%)	
Stage IV	13 (2.6%)	13 (2.6%)	
T stage, *n* (%)			0.002
T1	102 (20%)	66 (12.9%)	
T2	121 (23.7%)	155 (30.4%)	
T3	24 (4.7%)	23 (4.5%)	
T4	6 (1.2%)	13 (2.5%)	
N stage, *n* (%)			<0.001
N0	184 (36.7%)	146 (29.1%)	
N1	38 (7.6%)	57 (11.4%)	
N2	24 (4.8%)	50 (10%)	
N3	1 (0.2%)	1 (0.2%)	
M stage, *n* (%)			1.000
M0	165 (44.7%)	179 (48.5%)	
M1	12 (3.3%)	13 (3.5%)	
Smoker, *n* (%)			0.160
No	31 (6.2%)	43 (8.6%)	
Yes	219 (43.9%)	206 (41.3%)	
OS event, *n* (%)			0.001
Alive	181 (35.3%)	145 (28.3%)	
Dead	75 (14.6%)	112 (21.8%)	
Age, median (IQR)	66 (59, 73)	66 (59, 72)	0.652

**Table 2:** Univariate and multivariate Cox regression analyses of clinicopathologic features associated with OS. Abbreviations: HR: hazard ratio; CI: confidence interval.

Characteristics	Total (*N*)	Univariate analysis		Multivariate analysis	
		Hazard ratio (95% CI)	*P* value	Hazard ratio (95% CI)	*P* value
Age	494				
≤65	238	Reference			
>65	256	1.228 (0.915-1.649)	0.171		
Gender	504				
Female	270	Reference			
Male	234	1.060 (0.792-1.418)	0.694		
Pathologic stage	496				
Stage I and stage II	389	Reference			
Stage III and stage IV	107	2.624 (1.926-3.576)	<0.001	1.758 (0.802-3.852)	0.159
T stage	501				
T1 and T2	437	Reference			
T3 and T4	64	2.364 (1.621-3.448)	<0.001	1.835 (1.125-2.993)	0.015
N stage	492				
N0 and N1	419	Reference			
N2 and N3	73	2.300 (1.614-3.278)	<0.001	1.397 (0.677-2.884)	0.366
M stage	360				
M0	335	Reference			
M1	25	2.111 (1.232-3.616)	0.007	1.121 (0.499-2.517)	0.782
Smoker	490				
No	71	Reference			
Yes	419	0.887 (0.587-1.339)	0.568		
NDUFAF2	504				
Low	252	Reference			
High	252	1.453 (1.081-1.953)	0.013	1.538 (1.086-2.177)	0.015

**Table 3:** Gene sets enriched in the NDUFAF2 expression phenotype.

Gene set enrichment	Set size	Enrichment score	NES	*P* value	*P*.adjust	*q* values
REACTOME_G2_M_CHECKPOINTS	168	-0.51143	-1.63855	0.00102	0.028702	0.02278
REACTOME_DNA_REPLICATION	128	0.653243	3.010564	0.028571	0.130723	0.103754
REACTOME_DISEASES_OF_MITOTIC_CELL_CYCLE	36	0.48028	1.749202	0.011696	0.081309	0.064535
WP_METABOLIC_REPROGRAMMING_IN_COLON_CANCER	42	0.574395	2.154603	0.006369	0.067693	0.053727
WP_DNA_DAMAGE_RESPONSE	68	0.44162	1.823023	0.010989	0.081309	0.064535
WP_RETINOBLASTOMA_GENE_IN_CANCER	87	0.650934	2.846007	0.013333	0.085405	0.067786
BIOCARTA_CELLCYCLE_PATHWAY	23	0.537173	1.768104	0.013453	0.085478	0.067844
KEGG_CELL_CYCLE	124	0.482344	2.229059	0.02381	0.116588	0.092535
PID_P53_DOWNSTREAM_PATHWAY	136	0.336877	1.618717	0.028571	0.130723	0.103754
WP_GASTRIC_CANCER_NETWORK_2	31	0.433586	1.522692	0.042328	0.178819	0.141927

## Data Availability

The original contributions presented in the study are included in the article/Supplementary Material. Further inquiries can be directed to the corresponding authors.

## Supplementary Materials

Supplementary Materials Supplementary Figure 1: flow diagram of this study. Supplementary Figure 2: correlation analysis between NDUFAF2 and the cell cycle regulatory genes in LUAD in the TCGA database. (A) CCNA2, (B) CCNB1, (C) CCNB2, (D) CDC6, (E) CDC20, (F) CDC25A, (G) CDC25C, (H) CDC45, (I) CHEK1, (J) MCM2, (K) MCM6, (L) PCNA, (M) PLK1, (N) PTTG1, and (O) MCM4.
