# Supplementary figures and images for: Upregulation of NDUFAF2 in Lung Adenocarcinoma Is a Novel Independent Prognostic Biomarker
Source: Comput Math Methods Med. 2023 Jan 17;2023:2912968. doi: 10.1155/2023/2912968 (PMC9873462; doi:10.1155/2023/2912968)

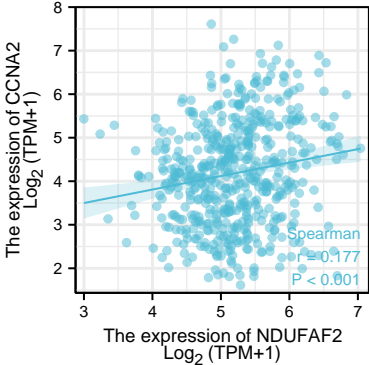

Supplement: Supplementary Materials — Supplementary Figure 1: flow diagram of this study. Supplementary Figure 2: correlation analysis between NDUFAF2 and the cell cycle regulatory genes in LUAD in the TCGA database. (A) CCNA2, (B) CCNB1, (C) CCNB2, (D) CDC6, (E) CDC20, (F) CDC25A, (G) CDC25C, (H) CDC45, (I) CHEK1, (J) MCM2, (K) MCM6, (L) PCNA, (M) PLK1, (N) PTTG1, and (O) MCM4. [file 2912968.f1.zip › CCNA2.pdf]

The expression of CCNB1  
 $\text{Log}_2(\text{TPM}+1)$

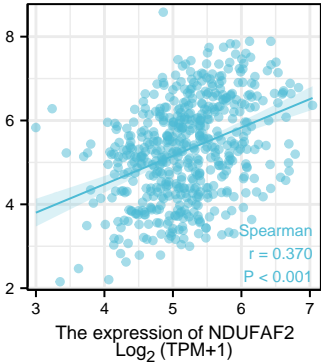

Supplement: Supplementary Materials — Supplementary Figure 1: flow diagram of this study. Supplementary Figure 2: correlation analysis between NDUFAF2 and the cell cycle regulatory genes in LUAD in the TCGA database. (A) CCNA2, (B) CCNB1, (C) CCNB2, (D) CDC6, (E) CDC20, (F) CDC25A, (G) CDC25C, (H) CDC45, (I) CHEK1, (J) MCM2, (K) MCM6, (L) PCNA, (M) PLK1, (N) PTTG1, and (O) MCM4. [file 2912968.f1.zip › CCNB1.pdf]

The expression of CCNB2  
 $\text{Log}_2(\text{TPM}+1)$

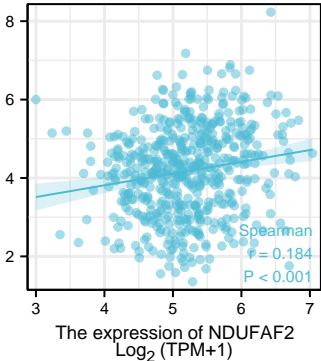

Supplement: Supplementary Materials — Supplementary Figure 1: flow diagram of this study. Supplementary Figure 2: correlation analysis between NDUFAF2 and the cell cycle regulatory genes in LUAD in the TCGA database. (A) CCNA2, (B) CCNB1, (C) CCNB2, (D) CDC6, (E) CDC20, (F) CDC25A, (G) CDC25C, (H) CDC45, (I) CHEK1, (J) MCM2, (K) MCM6, (L) PCNA, (M) PLK1, (N) PTTG1, and (O) MCM4. [file 2912968.f1.zip › CCNB2.pdf]

The expression of CDC20  
 $\text{Log}_2(\text{TPM}+1)$

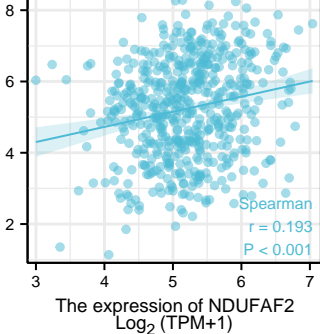

Supplement: Supplementary Materials — Supplementary Figure 1: flow diagram of this study. Supplementary Figure 2: correlation analysis between NDUFAF2 and the cell cycle regulatory genes in LUAD in the TCGA database. (A) CCNA2, (B) CCNB1, (C) CCNB2, (D) CDC6, (E) CDC20, (F) CDC25A, (G) CDC25C, (H) CDC45, (I) CHEK1, (J) MCM2, (K) MCM6, (L) PCNA, (M) PLK1, (N) PTTG1, and (O) MCM4. [file 2912968.f1.zip › CDC20.pdf]

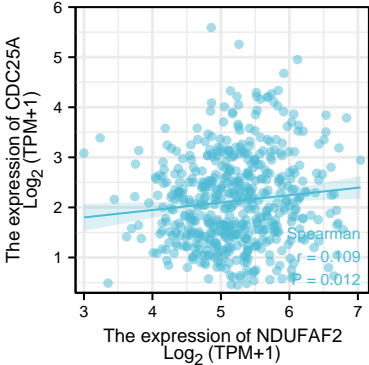

Supplement: Supplementary Materials — Supplementary Figure 1: flow diagram of this study. Supplementary Figure 2: correlation analysis between NDUFAF2 and the cell cycle regulatory genes in LUAD in the TCGA database. (A) CCNA2, (B) CCNB1, (C) CCNB2, (D) CDC6, (E) CDC20, (F) CDC25A, (G) CDC25C, (H) CDC45, (I) CHEK1, (J) MCM2, (K) MCM6, (L) PCNA, (M) PLK1, (N) PTTG1, and (O) MCM4. [file 2912968.f1.zip › CDC25A.pdf]

The expression of CDC25C  
 $\text{Log}_2(\text{TPM}+1)$

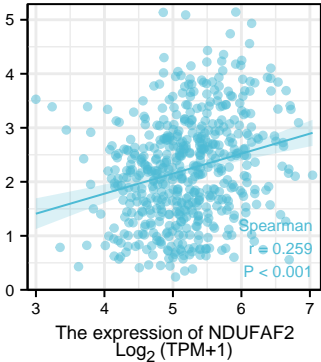

Supplement: Supplementary Materials — Supplementary Figure 1: flow diagram of this study. Supplementary Figure 2: correlation analysis between NDUFAF2 and the cell cycle regulatory genes in LUAD in the TCGA database. (A) CCNA2, (B) CCNB1, (C) CCNB2, (D) CDC6, (E) CDC20, (F) CDC25A, (G) CDC25C, (H) CDC45, (I) CHEK1, (J) MCM2, (K) MCM6, (L) PCNA, (M) PLK1, (N) PTTG1, and (O) MCM4. [file 2912968.f1.zip › CDC25C.pdf]

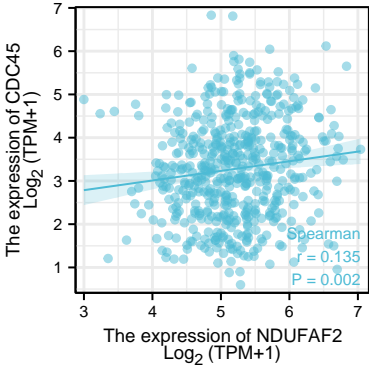

Supplement: Supplementary Materials — Supplementary Figure 1: flow diagram of this study. Supplementary Figure 2: correlation analysis between NDUFAF2 and the cell cycle regulatory genes in LUAD in the TCGA database. (A) CCNA2, (B) CCNB1, (C) CCNB2, (D) CDC6, (E) CDC20, (F) CDC25A, (G) CDC25C, (H) CDC45, (I) CHEK1, (J) MCM2, (K) MCM6, (L) PCNA, (M) PLK1, (N) PTTG1, and (O) MCM4. [file 2912968.f1.zip › CDC45.pdf]

The expression of CDC6  
 $\text{Log}_2(\text{TPM}+1)$

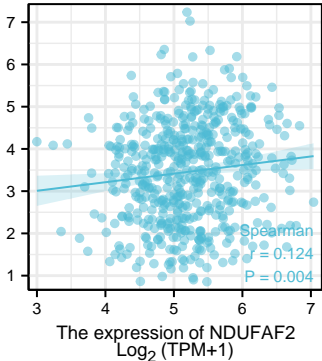

Supplement: Supplementary Materials — Supplementary Figure 1: flow diagram of this study. Supplementary Figure 2: correlation analysis between NDUFAF2 and the cell cycle regulatory genes in LUAD in the TCGA database. (A) CCNA2, (B) CCNB1, (C) CCNB2, (D) CDC6, (E) CDC20, (F) CDC25A, (G) CDC25C, (H) CDC45, (I) CHEK1, (J) MCM2, (K) MCM6, (L) PCNA, (M) PLK1, (N) PTTG1, and (O) MCM4. [file 2912968.f1.zip › CDC6.pdf]

The expression of CHEK1  
 $\text{Log}_2(\text{TPM}+1)$

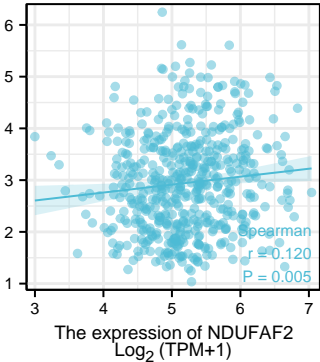

Supplement: Supplementary Materials — Supplementary Figure 1: flow diagram of this study. Supplementary Figure 2: correlation analysis between NDUFAF2 and the cell cycle regulatory genes in LUAD in the TCGA database. (A) CCNA2, (B) CCNB1, (C) CCNB2, (D) CDC6, (E) CDC20, (F) CDC25A, (G) CDC25C, (H) CDC45, (I) CHEK1, (J) MCM2, (K) MCM6, (L) PCNA, (M) PLK1, (N) PTTG1, and (O) MCM4. [file 2912968.f1.zip › CHEK1.pdf]

The expression of MCM2  
 $\text{Log}_2(\text{TPM}+1)$

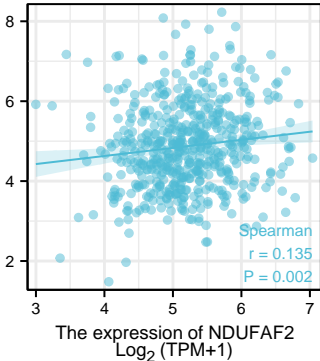

Supplement: Supplementary Materials — Supplementary Figure 1: flow diagram of this study. Supplementary Figure 2: correlation analysis between NDUFAF2 and the cell cycle regulatory genes in LUAD in the TCGA database. (A) CCNA2, (B) CCNB1, (C) CCNB2, (D) CDC6, (E) CDC20, (F) CDC25A, (G) CDC25C, (H) CDC45, (I) CHEK1, (J) MCM2, (K) MCM6, (L) PCNA, (M) PLK1, (N) PTTG1, and (O) MCM4. [file 2912968.f1.zip › MCM2.pdf]

The expression of MCM4  
 $\text{Log}_2(\text{TPM}+1)$

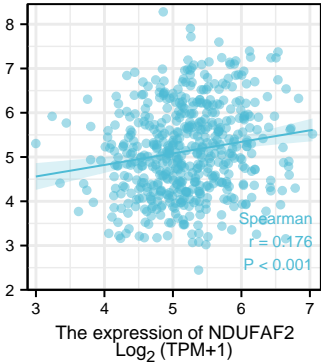

Supplement: Supplementary Materials — Supplementary Figure 1: flow diagram of this study. Supplementary Figure 2: correlation analysis between NDUFAF2 and the cell cycle regulatory genes in LUAD in the TCGA database. (A) CCNA2, (B) CCNB1, (C) CCNB2, (D) CDC6, (E) CDC20, (F) CDC25A, (G) CDC25C, (H) CDC45, (I) CHEK1, (J) MCM2, (K) MCM6, (L) PCNA, (M) PLK1, (N) PTTG1, and (O) MCM4. [file 2912968.f1.zip › MCM4.pdf]

The expression of MCM6  
 $\text{Log}_2(\text{TPM}+1)$

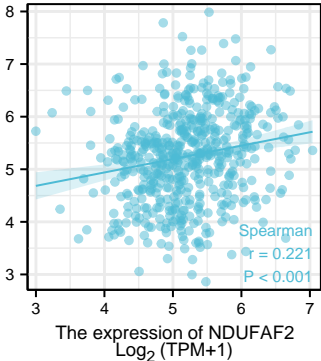

Supplement: Supplementary Materials — Supplementary Figure 1: flow diagram of this study. Supplementary Figure 2: correlation analysis between NDUFAF2 and the cell cycle regulatory genes in LUAD in the TCGA database. (A) CCNA2, (B) CCNB1, (C) CCNB2, (D) CDC6, (E) CDC20, (F) CDC25A, (G) CDC25C, (H) CDC45, (I) CHEK1, (J) MCM2, (K) MCM6, (L) PCNA, (M) PLK1, (N) PTTG1, and (O) MCM4. [file 2912968.f1.zip › MCM6.pdf]

The expression of PCNA  
 $\text{Log}_2(\text{TPM}+1)$

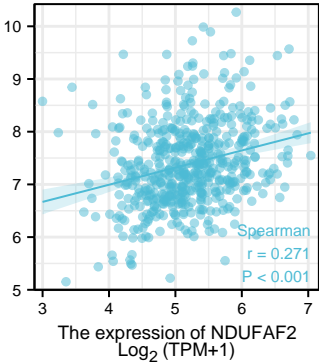

Supplement: Supplementary Materials — Supplementary Figure 1: flow diagram of this study. Supplementary Figure 2: correlation analysis between NDUFAF2 and the cell cycle regulatory genes in LUAD in the TCGA database. (A) CCNA2, (B) CCNB1, (C) CCNB2, (D) CDC6, (E) CDC20, (F) CDC25A, (G) CDC25C, (H) CDC45, (I) CHEK1, (J) MCM2, (K) MCM6, (L) PCNA, (M) PLK1, (N) PTTG1, and (O) MCM4. [file 2912968.f1.zip › PCNA.pdf]

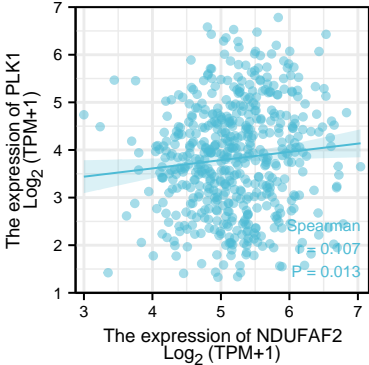

Supplement: Supplementary Materials — Supplementary Figure 1: flow diagram of this study. Supplementary Figure 2: correlation analysis between NDUFAF2 and the cell cycle regulatory genes in LUAD in the TCGA database. (A) CCNA2, (B) CCNB1, (C) CCNB2, (D) CDC6, (E) CDC20, (F) CDC25A, (G) CDC25C, (H) CDC45, (I) CHEK1, (J) MCM2, (K) MCM6, (L) PCNA, (M) PLK1, (N) PTTG1, and (O) MCM4. [file 2912968.f1.zip › PLK1.pdf]

The expression of PTTG1  
 $\text{Log}_2(\text{TPM}+1)$

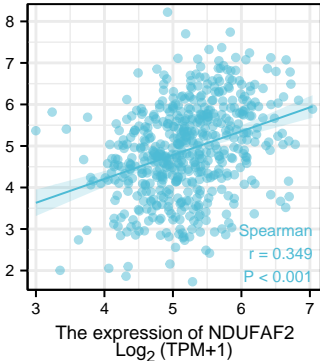

Supplement: Supplementary Materials — Supplementary Figure 1: flow diagram of this study. Supplementary Figure 2: correlation analysis between NDUFAF2 and the cell cycle regulatory genes in LUAD in the TCGA database. (A) CCNA2, (B) CCNB1, (C) CCNB2, (D) CDC6, (E) CDC20, (F) CDC25A, (G) CDC25C, (H) CDC45, (I) CHEK1, (J) MCM2, (K) MCM6, (L) PCNA, (M) PLK1, (N) PTTG1, and (O) MCM4. [file 2912968.f1.zip › PTTG1.pdf]

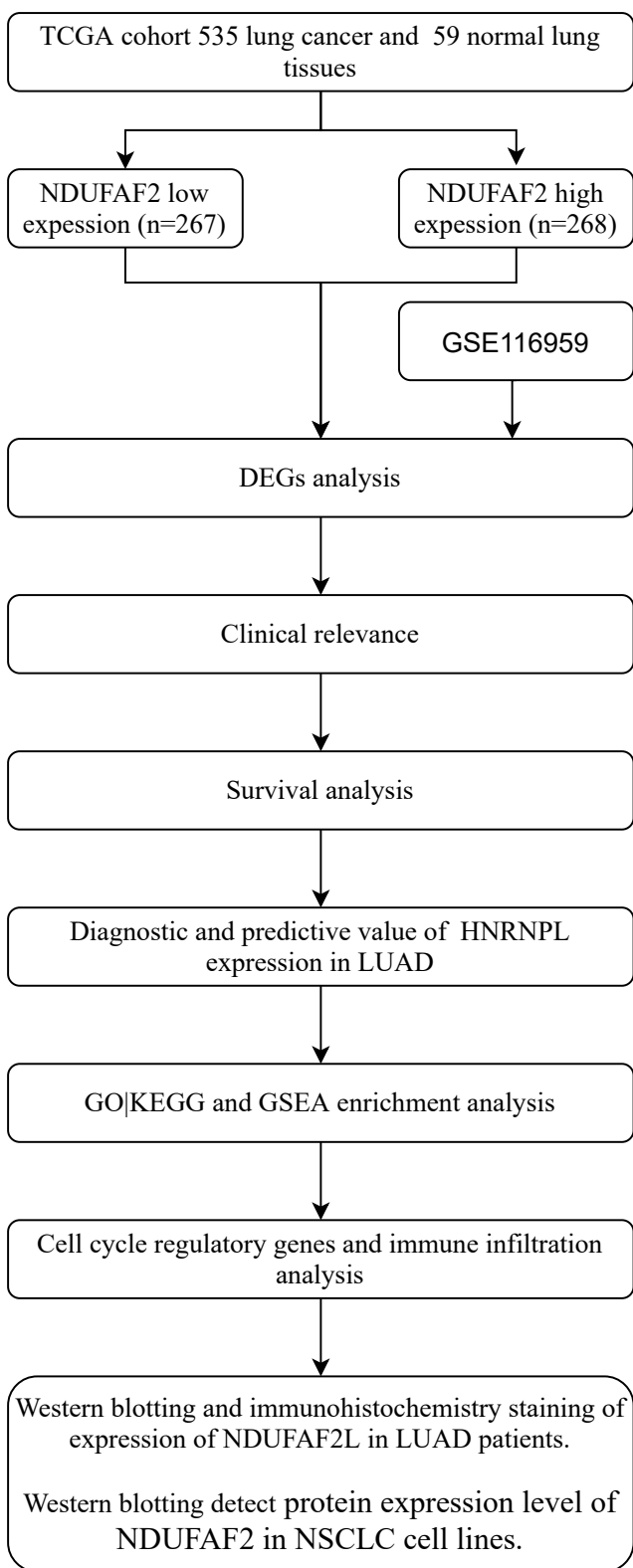

Supplement: Supplementary Materials — Supplementary Figure 1: flow diagram of this study. Supplementary Figure 2: correlation analysis between NDUFAF2 and the cell cycle regulatory genes in LUAD in the TCGA database. (A) CCNA2, (B) CCNB1, (C) CCNB2, (D) CDC6, (E) CDC20, (F) CDC25A, (G) CDC25C, (H) CDC45, (I) CHEK1, (J) MCM2, (K) MCM6, (L) PCNA, (M) PLK1, (N) PTTG1, and (O) MCM4. [file 2912968.f1.zip › Supplementary figure 1 (1).pdf]
